# Native High Density Lipoproteins (HDL) Interfere with Platelet Activation Induced by Oxidized Low Density Lipoproteins (OxLDL)

**DOI:** 10.3390/ijms140510107

**Published:** 2013-05-10

**Authors:** Sigrun Badrnya, Alice Assinger, Ivo Volf

**Affiliations:** 1 Institute of Physiology-Centre for Physiology & Pharmacology, Medical University of Vienna; Schwarzspanierstr. 17, 1090 Vienna, Austria; E-Mails: sigrun.badrnya@meduniwien.ac.at (S.B.); alice.assinger@meduniwien.ac.at (A.A.); 2 Department of Molecular Medicine, Solna, Karolinska Institutet, 17177 Stockholm, Sweden

**Keywords:** platelets, reactive oxygen species, oxidized low density lipoproteins, high density lipoproteins, inflammation, CD40 ligand

## Abstract

Platelets and lipoproteins play a crucial role in atherogenesis, in part by their ability to modulate inflammation and oxidative stress. While oxidized low density lipoproteins (OxLDL) play a central role in the development of this disease, high density lipoproteins (HDL) represent an atheroprotective factor of utmost importance. As platelet function is remarkably sensitive to the influence of plasma lipoproteins, it was the aim of this study to clarify if HDL are able to counteract the stimulating effects of OxLDL with special emphasis on aspects of platelet function that are relevant to inflammation. Therefore, HDL were tested for their ability to interfere with pro-thrombotic and pro-inflammatory aspects of platelet function. We are able to show that HDL significantly impaired OxLDL-induced platelet aggregation and adhesion. In gel-filtered platelets, HDL decreased both the formation of reactive oxygen species and CD40L expression. Furthermore, HDL strongly interfered with OxLDL-induced formation of platelet-neutrophil aggregates in whole blood, suggesting that platelets represent a relevant and sensitive target for HDL. The finding that HDL effectively competed with the binding of OxLDL to the platelet surface might contribute to their atheroprotective and antithrombotic properties.

## 1. Introduction

Atherosclerosis, resulting in cardiovascular disease (CVD) and its thrombotic consequences represents the major source of morbidity and mortality in the Western world.

Oxidative stress and inflammation are regarded as central and causally linked features of the atherosclerotic process [1]. The observation that macrophages are transformed into foam cells due to unrestricted cellular uptake of oxidatively modified LDL (OxLDL) represented initial evidence for a potential involvement of OxLDL in this process and generated considerable interest regarding the role of lipoproteins and oxidative stress in atherogenesis [1]. Subsequent studies were able to demonstrate an atherogenic impact of OxLDL on virtually all cell types that are relevant to the development of this disease [1]. Furthermore, the *in vivo* occurrence of OxLDL has been confirmed both in atherosclerotic lesions as well as in plasma (reviewed in [1,2]).

As thrombotic events are responsible for the lethal consequences of atherosclerosis, the central importance of blood platelets to this disease has been recognized for many years. Nevertheless, recent findings clearly demonstrate that (activated) platelets also play a key role in the development of atherosclerosis, as stimulated platelets are causally involved in the onset of inflammatory reactions, cell proliferation and immune responses [3]. Platelets represent very sensitive sentinels in the blood that are known to be activated by oxidative stress and inflammation. Therefore modulation of platelet activation appears of central importance also in the context of inflammation-related disease.

High density lipoproteins (HDL), in part by acting as an antioxidant, represent an atheroprotective factor of utmost importance. High plasma levels of HDL have been shown in several epidemiological studies to be atheroprotective [4,5] while low HDL concentrations relate to endothelial dysfunction and increased LDL oxidation in otherwise healthy young men [6].

The perhaps most prominent feature of HDL lies in its ability to mediate reverse cholesterol transport [7], *i.e.*, the transport of excess cholesterol from the periphery to the liver for bile excretion. Nevertheless, HDL possess a number of functions distinct from reverse cholesterol transport that are clearly beneficial in the context of atherosclerotic disease. For example, HDL enhance synthesis and bioavailability of nitric oxide [8] and they possess anti-oxidative as well as anti-inflammatory properties [9], in part by modulating the function of various cell types that are involved in the atherosclerotic process.

Of particular interest, HDL have been reported to effectively counteract some of the pro-atherosclerotic effects of OxLDL in several cell types: HDL have been shown to antagonize vasoconstriction in response to OxLDL [10] as well as neutrophil respiratory burst [11]. In endothelial cells, HDL were found to inhibit OxLDL-induced expression of Monocyte Chemotactic Protein 1 [12] as well as overexpression of both Intracellular Adhesion Molecule 1 and Vascular Cell Adhesion Protein 1 [13].

Regarding potential protective effects of HDL in the context of OxLDL-mediated platelet activation, only limited information is available [14,15]. As platelet function is known to be remarkable sensitive to the influence of plasma lipoproteins and as OxLDL represent a potent platelet agonist [16], it was the aim of this study to clarify if HDL are able to counteract the stimulating effects of OxLDL with special emphasis on aspects of platelet function that are relevant to inflammation.

## 2. Results and Discussion

### 2.1. Native HDL Counteract Hyp-OxLDL-Induced Platelet Aggregation and Adhesion

While native HDL (nHDL) have been reported to interfere with platelet activation induced by ADP and thrombin, there is only limited information available with regard to platelet activation induced by hypochlorite-oxidized LDL (hyp-OxLDL) [14,15]. The modification of LDL by hypochlorite is of special relevance as hypochlorous acid/hypochlorite is formed by the enzyme myeloperoxidase (MPO) and both MPO as well as hypochlorite-modified (lipo)protein has been detected in human atherosclerotic lesions [17]. Furthermore, platelet-activating properties of hypochlorite-oxidized LDL differ from that of (commonly used) trace metal-oxidized LDL [18].

**Figure 1:**
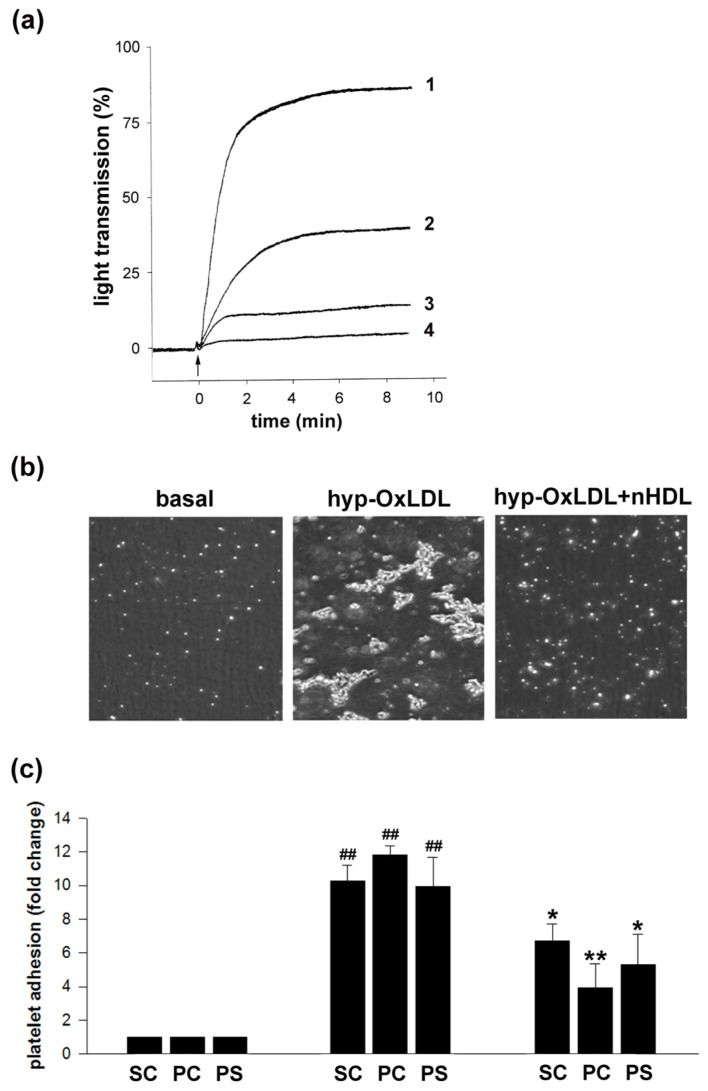
Effects of native HDL (nHDL) on hypochlorite-oxidized LDL (hyp-OxLDL)-induced platelet aggregation and platelet adhesion. (**a**) Platelet aggregation induced by 50 μg/mL hyp-OxLDL after pretreatment with nHDL of different concentrations: (1) control (without nHDL), (2) 50 μg/mL nHDL, (3) 200 μg/mL nHDL, (4) 400 μg/mL nHDL. Tracings of one typical experiment out of eight experiments. (**b**,**c**) Platelet adhesion to a collagen-coated surface under high shear rates (1000 s^−1^): Representative phase-contrast microscopic images (**b**) and quantification of relative surface coverage (SC), particle count (PC) and particle size (PS) (**c**). Basal adhesion, adhesion after incubation of platelets with hyp-OxLDL (100 μg/mL) and after pretreatment with nHDL (400 μg/mL) prior to hyp-OxLDL (100 μg/mL) stimulation. Means ± SD of three experiments. Significances relative to control (^#^) or relative to hyp-OxLDL-stimulated samples (*). * *p* < 0.05; ^##,^** *p* < 0.01.

To determine whether nHDL are able to counteract hyp-OxLDL-induced platelet activation, we first analyzed the ability of nHDL to interfere with platelet aggregation. As depicted in Figure 1a, nHDL dose-dependently attenuated hyp-OxLDL-induced platelet aggregation. Although determination of platelet aggregation is still considered the “gold standard” of platelet function tests, a drawback of light transmittance aggregometry lies in its insensitivity to detect the formation of small aggregates. Therefore, to study the effects of nHDL on hyp-OxLDL-induced aggregate formation in more detail, we used a flow model where platelets (in the presence and absence of nHDL) were incubated with hyp-OxLDL and were subsequently perfused over a collagen-coated surface at a shear rate of 1000 s^−1^. We found that hyp-OxLDL significantly increased platelet adhesion to collagen (10-fold increase in surface coverage and 12-fold increase in particle count) and the formation of platelet-platelet aggregates (10-fold increase in particle size), which is in principal agreement with findings recently obtained with trace metal-oxidized LDL [19]. As depicted in Figure 1b and Figure 1c, pretreatment of platelets with nHDL prior to stimulation with hyp-OxLDL resulted in both a significantly decreased number of particles that adhered to the collagen surface and reduced particle size. However, platelet aggregates of up to 5 platelets were still detectable in the presence of nHDL.

### 2.2. Native HDL Attenuate Intraplatelet ROS Formation and CD40L Expression Induced by Hyp-OxLDL

As reactive oxygen species (ROS) play an important role in the course of platelet activation [20], we next determined the impact of nHDL on the hyp-OxLDL-mediated formation of ROS within platelets.

**Figure 2:**
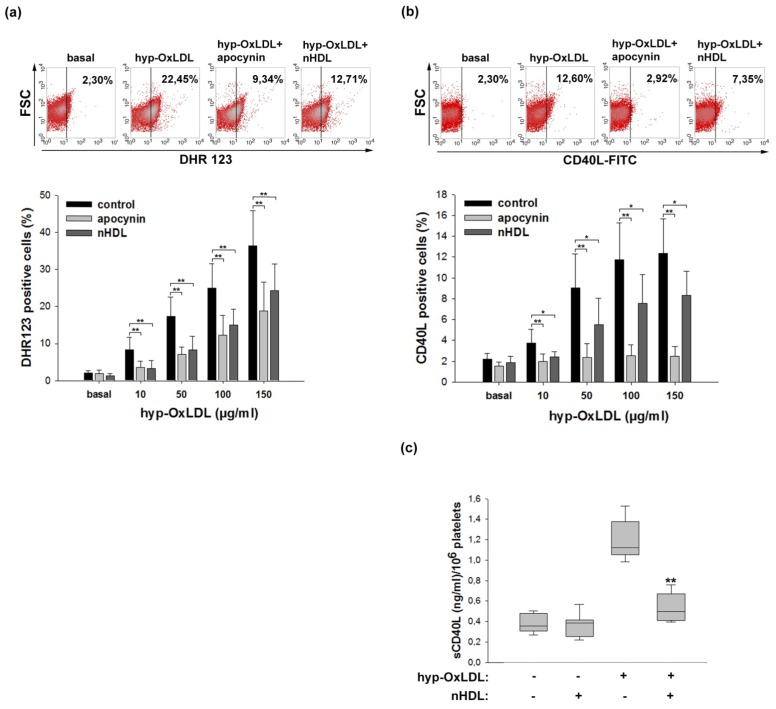
Impact of nHDL on hyp-OxLDL-induced intraplatelet ROS formation and surface expression and release of CD40L. (**a**,**b**) Effects of nHDL (200 μg/mL) and apocynin (500 μM) on intraplatelet ROS generation (**a**) and surface expression of CD40L (**b**) in response to the indicated concentrations of hyp-OxLDL Upper panel: Representative dot plots obtained with 100 μg/mL hyp-OxLDL; lower panel: Means ± SD of 8 experiments. * *p* < 0.05; ** *p* < 0.01. (**c**) Determination of soluble CD40L in releasates from isolated platelets stimulated with hyp-OxLDL (100 μg/mL) in the absence and presence of nHDL (400 μg/mL) for 4 h. Means ± SD of eight experiments. ** *p* < 0.01.

As shown in Figure 2a, hyp-OxLDL dose-dependently induced intraplatelet formation of ROS and nHDL significantly interfered with this process. Effects of nHDL were more pronounced at lower agonist concentrations, in agreement with data recently reported for the strong platelet agonist thrombin [21]. Interestingly, modulating effects of nHDL on ROS formation have also been described in neutrophils where these lipoproteins interfere with respiratory burst activation in response to OxLDL, but not with that induced by formyl-methionyl-leucyl-phenylalanin and zymosan [11]. Furthermore, findings obtained with human polymorphonuclear leukocytes indicate that T cell contact-mediated activation of respiratory burst is also susceptible to the action of HDL [22].

As also shown in Figure 2a, NAD(P)H-oxidase inhibitor, apocynin, significantly interfered with platelet ROS formation induced by hyp-OxLDL. This is of special relevance as the formation of NAD(P)H-oxidase-derived ROS represents a prerequisite for surface expression (and subsequent liberation) of CD40L [23] and the interaction between CD40L (a member of the tumor necrosis factor alpha family) and its receptor CD40 constitutes an integral part of the inflammatory pathway in the vascular system [24]. Furthermore, platelets represent the main source of CD40L as more than 95% of the circulating CD40L exists in platelets [25].

In line with the demonstrated interrelation between ROS and CD40L, we found that nHDL were able to reduce surface expression of CD40L in a statistically significant way at all hyp-OxLDL concentrations tested (Figure 2b). While expression of CD40L induced by low concentrations of hyp-OxLDL was attenuated by nHDL to an extent comparable to that observed by apocynin, the inhibitory effect of nHDL in the presence of higher concentrations of hyp-OxLDL (>50 μg/mL) was less pronounced, but still statistically significant (Figure 2b).

The finding that inhibitory effects of apocynin on intraplatelet ROS production are less pronounced than those on CD40L expression probably indicates a contribution of other enzymatic sources to ROS production induced by hyp-OxLDL. Results obtained from quantification of soluble CD40L (depicted in Figure 2c) are in line with those obtained from quantification of platelet-bound CD40L, thereby ruling out the possibility that decreased levels of platelet-bound CD40L that are observed in the presence of nHDL might arise from enhanced cleavage of CD40L.

From these data we conclude that nHDL are able to interfere with both hyp-OxLDL-induced intraplatelet ROS formation and surface expression of CD40L.

### 2.3. Native HDL Modulate Expression of CD62P and Formation of Platelet-Neutrophil Aggregates

In response to distinct stimuli, platelets translocate P-selectin from alpha granules to the cell surface, where this protein functions as a cell adhesion molecule, mediating the physical association of platelets with leukocytes and endothelial cells.

**Figure 3:**
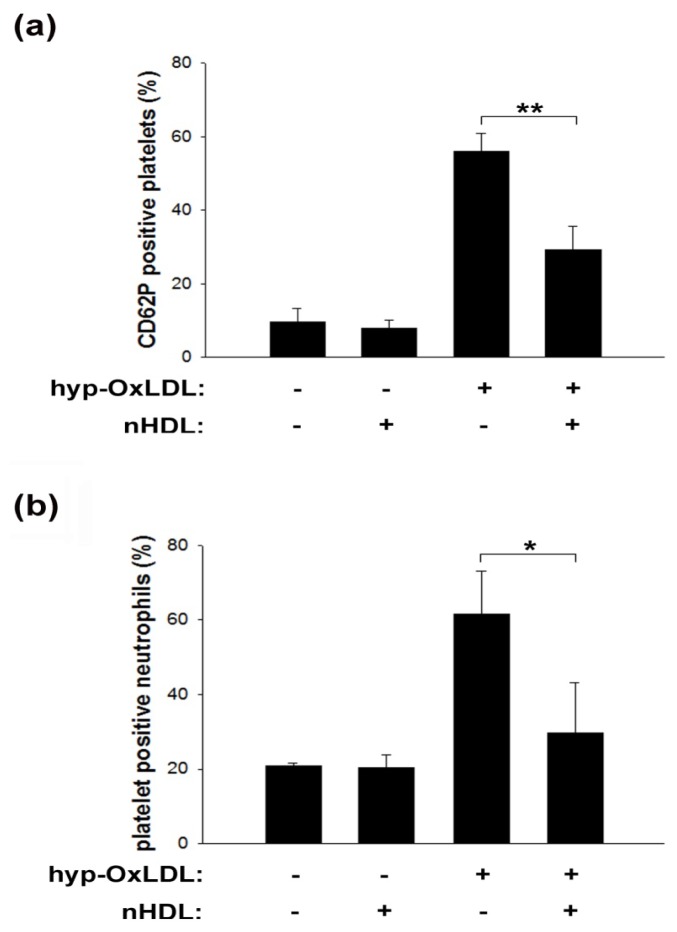
Effects of nHDL on hyp-OxLDL-induced surface expression of CD62P and formation of platelet-neutrophil aggregates. (**a**) Influence of nHDL (200 μg/mL) on surface expression of P-selectin induced by hyp-OxLDL (100 μg/mL). Means ± SD of nine experiments. ** *p* < 0.01; (**b**) Influence of nHDL (200 μg/mL) on the formation of platelet-neutrophil aggregates induced by hyp-OxLDL (100 μg/mL) in whole blood. Means ± SD of four experiments. * *p* < 0.05.

In light of the general relevance of such interactions especially in the context of inflammation [26,27], we next studied if nHDL might interfere with platelet P-selectin expression. Indeed, nHDL significantly reduced hyp-OxLDL-induced expression of CD62P on the surface of isolated platelets by approximately 50% (depicted in Figure 3a).

The formation of platelet-neutrophil aggregates (PNA) represents a sensitive indicator for platelet activation that primarily depends on platelet surface P-selectin binding to its counterreceptor PSGL-1 on the leukocyte surface [28]. We recently showed that hyp-OxLDL mediate the formation of PNA in whole blood, thereby facilitating neutrophil adhesion to an endothelial monolayer and subsequent transmigration [29]. Here we are able to show that PNA formation induced by hyp-OxLDL in whole blood significantly decreased in the presence of nHDL (Figure 3b).

This finding is of special interest as increased transmigration of neutrophils, due to the formation of platelet-neutrophil aggregates, might result in weakening of the atherosclerotic plaque und subsequent plaque rupture *in vivo* [30]. Platelet-leukocyte interactions are crucial for several other (patho)physiologic events *in vivo* [26]. These interactions take place at the site of ruptured plaques [31] and the presence of PNA is associated with acute myocardial infarction [32].

Our findings that HDL interfere with platelet-neutrophil interaction extend findings of a previously published study [33] that focused on modulating effects of HDL on lipopolysaccharide- and phorbol-myristate-acetate-mediated activation of isolated leukocytes. In light of our recent findings [29] and the conditions employed for the respective experiments, we are able to mainly attribute PNA formation to the effects of hyp-OxLDL on platelets. However, consequences of platelet-leukocyte interaction are complex as activated leukocytes enhance platelet aggregation and thromboxane release [34] while binding of activated platelets to resting leukocytes results in leukocyte activation [35,36]. Therefore, we cannot completely rule out that the observed decrease in PNA formation by nHDL, to some extent, might also be caused by an nHDL-mediated decrease in neutrophil activation. However, our data indicate that platelets represent the main target of nHDL.

Notably, the finding that lipoprotein-mediated effects on platelet function can be observed in whole blood, *i.e.*, persist in a complex and biologically relevant environment (including other cell types, plasma proteins and antioxidants), appears of pivotal importance to estimate the potential *in vivo* relevance of the obtained findings.

### 2.4. Native HDL Compete with Binding of Hyp-OxLDL to Human Platelets

As hyp-OxLDL-mediated platelet activation has been shown to be a consequence of specific lipoprotein binding to platelet scavenger receptor CD36 (and at least one other receptor) [19,37–39] and as HDL are also a recognized ligand of CD36 [40], we tested the hypothesis that HDL might interfere with binding of hyp-OxLDL to the platelet surface.

**Figure 4:**
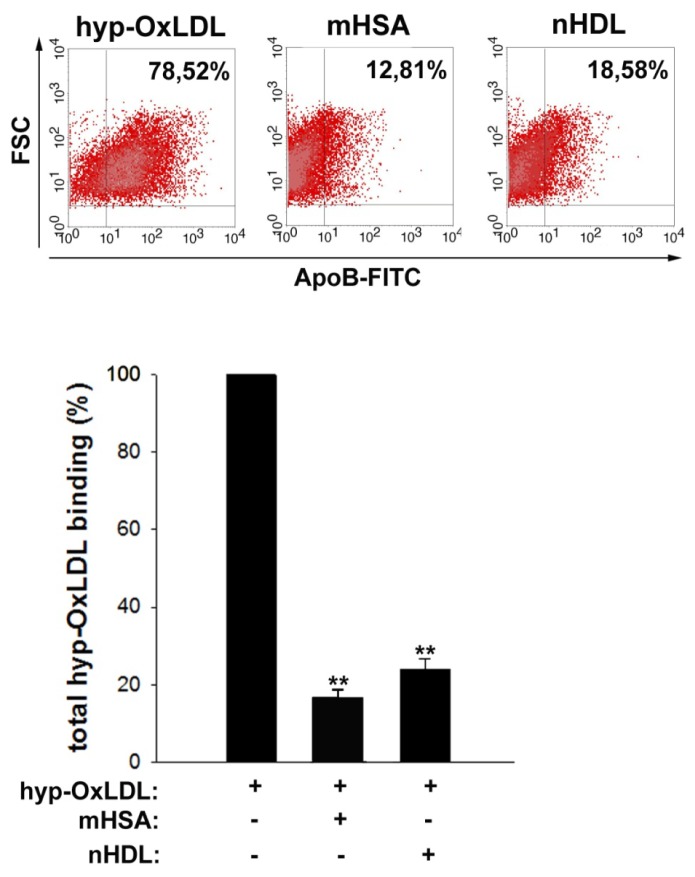
Effects of nHDL on platelet binding of hyp-OxLDL. Binding of hyp-OxLDL (50 μg/mL) to platelets was determined in the presence of mHSA (500 μg/mL) and nHDL (400 μg/mL). Representative dot plots and means ± SD of eight experiments. ** *p* < 0.01.

In light of the strong platelet-activating properties of hyp-OxLDL, these experiments were performed at 4 degrees [39,41] to avoid artefacts resulting from platelet aggregation or potential cellular lipoprotein uptake. We were able to confirm specific and saturable binding of both hyp-OxLDL and nHDL to the platelet membrane (not shown). As depicted in Figure 4, nHDL strongly interfered with hyp-OxLDL binding to the platelet surface, to an extent that was roughly comparable to that achieved by the scavenger receptor antagonist maleylated human serum albumin (total binding of hyp-OxLDL in the presence of nHDL: 23.9% ± 2.9%, in the presence of mHSA: 16.6% ± 2.2%).

These findings indicate that HDL are able to compete for a significant portion of hyp-OxLDL binding to the platelet surface. Therefore, the ability of HDL to interfere with hyp-OxLDL-mediated platelet activation might at least in part relate to its ability to reduce hyp-OxLDL binding to the cell surface.

However, it is important to note that the mechanisms by which HDL interfere with platelet activation also represent the consequence of specific signaling [21]. (Subfractions of) HDL have been shown to induce nitric oxide synthesis within platelets [42] and stimulation of protein kinase C [43]. Accordingly, HDL have been shown to interfere with platelet activation induced by (low doses of) ADP and thrombin [21,44]. While these effects have been attributed to the protein moiety of HDL, recently obtained findings indicate that negatively charged phospholipids within HDL can also interfere with both the binding of HDL_3_ to the platelet surface and with thrombin-induced platelet activation and that scavenger receptor BI (SR-BI) is essential for these effects [44]. Although there are convincing data that platelet-activating properties of hyp-OxLDL are mediated by scavenger receptor CD36 rather than SR-BI [39], these findings are not in conflict with our results as the ability to bind a broad array of different ligands (including lipoproteins and negatively charged phospholipids) is a common feature of class B scavenger receptors (*i.e*., of both CD36 and SR-BI). Therefore, phospholipids within the lipoproteins might contribute to the ability of HDL to compete with binding of hyp-OxLDL to the platelet surface.

As semi-stable chloramines are an integral constituent of hyp-OxLDL, chloramine breakup during the performed experiments and subsequent radical formation might to some extent alter biochemical and functional properties of both hyp-OxLDL and HDL. While we cannot completely exclude the contribution of such mechanisms, the finding that platelet activation by hyp-OxLDL is impaired by HDL, but not by albumin (see Figure S1) rules out the possibility that a decay of chloramines within hyp-OxLDL due to temperature or transfer to amino groups residing on other (lipo)proteins weakens the platelet activating properties of hyp-OxLDL. Moreover, binding and competition experiments were performed under conditions where chloramines are stable.

## 3. Experimental Section

### 3.1. Blood Collection and Isolation of Human Platelets

Informed consent was obtained from all volunteers in accordance with the approval of the Human Ethics Committee of the Medical University of Vienna (EK237/2004). Blood donors were apparently healthy and free of any anti-platelet medication for at least two weeks. Blood drawn with a 20-G needle was collected into tubes with 3.8% (*w*/*v*) trisodium citrate and immediately centrifuged at 125× *g* for 20 min at room temperature (RT) to obtain platelet-rich plasma (PRP). Only the upper two thirds of the PRP fraction were taken to avoid contamination with other cell types. Platelets were isolated by centrifugation (3000× *g*, 2 min) in the presence of PGI_2_ (1 μM; Sigma-Aldrich, Vienna, Austria) or gel filtered on a Sepharose 4B column with HEPES-Tyrode buffer containing 0.5% human serum albumin as described previously [29,45]. All experiments were performed with isolated platelets, unless otherwise stated. All concentrations are given as final concentrations.

### 3.2. Isolation of Lipoproteins and Oxidative Modification of LDL

Isolation and oxidative modification of lipoproteins was performed as described previously [16]. Plasma and lipoproteins were kept at 4 °C to reduce inadvertent oxidative modification. Briefly, lipoproteins were isolated from plasma of healthy donors by density gradient ultracentrifugation, filtered (0.45 μm) and subsequently rebuffered by size exclusion chromatography using EconoPac 10DG columns (Bio-Rad Laboratories, Hercules, CA, USA). Oxidative modification of LDL was achieved by a 400-fold molar excess of hypochlorite over LDL, resulting in oxidatively modified lipoproteins that displayed a relative electrophoretic mobility of 1.95–2.0 (compared to native LDL). In accordance with the reported lack of lipid peroxidation products after hypochlorite modification, the amount of thiobarbituric acid-reactive substances within hyp-OxLDL was always below 1 nmol/mg [16]. Lipoprotein concentrations are expressed in terms of their protein content as determined using the Lowry protocol [46]. Lipoproteins from 8 healthy donors were used for the presented experiments.

### 3.3. Platelet Adhesion under Shear Stress

Washed platelets were preincubated with HDL (400 μg/mL) for 5 min or left untreated before stimulation with hyp-OxLDL (100 μg/mL) for 2 min. Subsequently, platelets were diluted in PBS (1:10), perfused over collagen IV coated channel slides (iBiDi, Martinsried, Germany) for another 2 min and monitored by real-time capturing of microscopic phase contrast images. The flow rate was set to 5 mL/min resulting in a shear rate of approximately 1000 s^−1^. After a 5 min washout with PBS, images were taken in 5 separate fields along the centerline of the channel and analyzed using ImagePro Software (Version 5.0, DataCell, Finchampstead, United Kingdom, 2003) and ImageJ (Version 1.45s, Wayne Rasband, NIH, Bethesda, MD, USA, 2012).

### 3.4. Aggregation

Platelet aggregation induced by hyp-OxLDL (50 μg/mL) was monitored after pretreatment of platelets with nHDL (50, 200, 400 μg/mL or buffer) for 5 min in an optical 4-channel aggregometer (490-4D, Chronolog Corp., Havertown, PA, USA) exactly as described previously [16].

### 3.5. Quantification of CD62P

After preincubation of isolated platelets with nHDL (200 μg/mL) or human serum albumin (HSA; 50–400 μg/mL) for 5 min, followed by stimulation with hyp-OxLDL (100 μg/mL) for 10 min, platelets were incubated with PE-labelled anti CD62P mAb (BD, Schwechat, Austria) for 30 min in the dark. After fixation with 1% formaldehyde (Sigma-Aldrich, Vienna, Austria), platelets were analyzed by flow cytometry.

### 3.6. Quantification of CD40L Surface Expression and Reactive Oxygen Species (ROS)

Gel-filtered platelets were either preincubated for 5 min with nHDL (200 μg/mL), apocynin (500 μM), HSA (50–400 μg/mL) or left untreated before stimulation with hyp-OxLDL (10–150 μg/mL) for 10 min. Platelets were then either incubated with FITC-labelled anti CD40L mAb (BD, Schwechat, Austria) for 30 min or dihydrorhodamine 123 (DHR 123; 10 μM, Enzo Life Sciences, Lausen, Switzerland) for 10 min in the dark. Subsequently, cells were fixed (1% formaldehyde) and immediately analyzed by flow cytometry.

### 3.7. Quantification of Soluble CD40L (sCD40L)

Isolated platelets were either pretreated for 5 min with nHDL (400 μg/mL) or left untreated prior to stimulation with hyp-OxLDL (100 μg/mL). After 4 h of incubation at RT, platelets were centrifuged at 3000× *g* for 1.5 min and the supernatant was stored at −80 °C. Levels of sCD40L in the supernatant were determined by a high sensitivity ELISA (Bender MedSystems, Vienna, Austria) according to the manufacturer’s instructions.

### 3.8. Determination of Platelet-Neutrophil Aggregates

Formation of platelet-neutrophil-aggregates (PNA) was measured in whole blood by two-colour flow cytometry as described previously [47]. Briefly, whole blood was either pretreated with nHDL (200 μg/mL) for 5 min of left untreated prior to stimulation with hyp-OxLDL (100 μg/mL) for 10 min and incubated with platelet-specific anti CD61-Alexa647 (BioLegend, San Diego, CA, USA) and leukocyte-specific anti CD45-FITC mAb (BioLegend, San Diego, CA, USA) for 20 min in the dark. After fixation with 0.5% formaldehyde the percentage of platelet-bound neutrophils was quantified as CD45/CD61 double-positive neutrophils.

### 3.9. Binding Studies

Binding experiments of hyp-OxLDL to the platelet surface were performed as described previously [39]. Briefly, platelets were pre-chilled on ice for 30 min, then mHSA (500 μg/mL) or nHDL (400 μg/mL) were added and after 30 min platelets were incubated with hyp-OxLDL (50 μg/mL) at 4 °C for another 2 h. Thereafter platelets were fixed with 1% formaldehyde and incubated with anti-ApoB mAb (Rockland, Gilbertsville, PA, USA) for 30 min. After centrifugation (1000× *g*, 1.5 min) and resuspension in PBS, platelets were incubated with Alexa 488-labelled anti-goat antibody (Invitrogen, Vienna, Austria) for 45 min and analyzed by flow cytometry.

Flow cytometric analyses were performed using a FACSCalibur flow cytometer (BD, Schwechat, Austria).

### 3.10. Statistical Analysis

Flow cytometric data are presented as means ± standard deviation (SD). Statistical analyses were performed with GraphPad Prism 5 for Windows (Version 5.01, GraphPad Software, San Diego, CA, USA, 2007), using analysis of variance (ANOVA) followed by a Bonferroni *post hoc* test for effects of multiple treatments. Comparisons of individual treatments were tested by an unpaired *t-*test (two-tailed). ELISA data are depicted as box plots indicating the median, 1st and 3rd Quartile. Graphics of the calculated data were drawn with Sigma Plot 10.0. * *p* values < 0.05 indicate a statistically significant difference; **, ## *p* values < 0.01 indicate a statistically very significant difference.

## 4. Conclusions

Our findings that HDL not only interfere with pro-thrombotic aspects of platelet function, but also with OxLDL-induced intraplatelet ROS formation as well as the exposure of CD40L and the formation of platelet-neutrophil aggregates significantly broaden the array of identified anti-inflammatory and atheroprotective properties of HDL.

Platelets appear to represent a relevant and sensitive target for HDL. As oxidized LDL are a potent platelet agonist and play a key role in the development of atherosclerosis and (other types of) inflammatory disease, platelets might also represent a clinically relevant target for treatment of patients with reconstituted HDL that was recently shown to result in favorable changes in human atherosclerotic plaque [48].

## Supplementary Information
