# Supplementary material for: Native High Density Lipoproteins (HDL) Interfere with Platelet Activation Induced by Oxidized Low Density Lipoproteins (OxLDL)
Source: Int J Mol Sci. 2013 May 10;14(5):10107–21. doi: 10.3390/ijms140510107 (PMC3676831; doi:10.3390/ijms140510107)
Supplement: Supplementary file 1 [file ijms-14-10107-s001.pdf]

Supplementary Information

Figure S1. Effect of human serum albumin (HSA; 50–400 µg/mL) on surface expression of CD40L (**a**) and CD62P (**b**) induced by hyp-OxLDL (100 µg/mL). Means ± SD of 6 experiments.

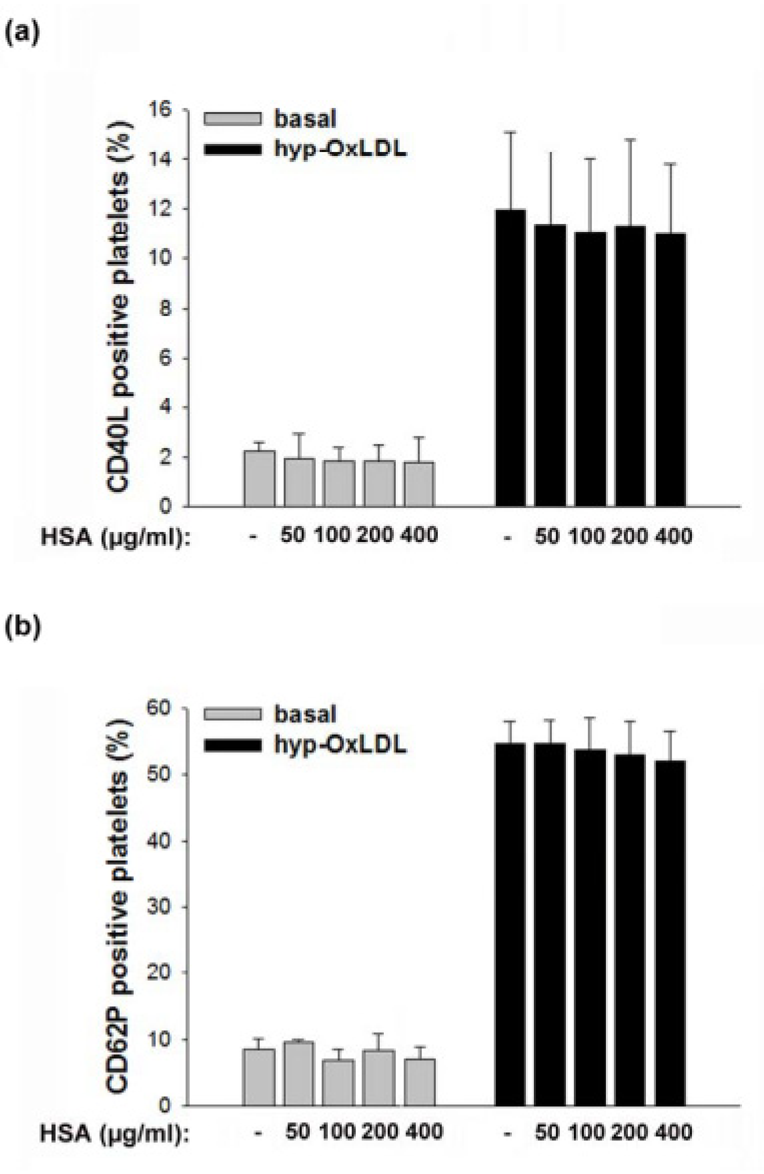

© 2013 by the authors; licensee MDPI, Basel, Switzerland. This article is an open access article distributed under the terms and conditions of the Creative Commons Attribution license (http://creativecommons.org/licenses/by/3.0/).
